# Tuberculosis-associated mortality in Shanghai, China: a longitudinal study

**DOI:** 10.2471/BLT.15.154161

**Published:** 2015-09-28

**Authors:** Weibing Wang, Qi Zhao, Zhengan Yuan, Yihui Zheng, Yixing Zhang, Liping Lu, Yun Hou, Yue Zhang, Biao Xu

**Affiliations:** a Department of Epidemiology, School of Public Health and Key Laboratory of Public Health Safety (Ministry of Education), Fudan University, 138 Yi Xue Yuan Road, Shanghai 200032, China.; b Shanghai Center for Disease Control and Prevention, Shanghai, China.; c Putuo District Center for Disease Control and Prevention, Shanghai, China.; d Pudong District Center for Disease Control and Prevention, Shanghai, China.; e Songjiang District Center for Disease Control and Prevention, Shanghai, China.; f Yangpu District Center for Disease Control and Prevention, Shanghai, China.

## Abstract

**Objective:**

To determine excess mortality in a cohort of people with tuberculosis in Shanghai.

**Methods:**

Participants were local residents in 4 (of 19) districts in Shanghai, registered in one of four tuberculosis clinics between January 1, 2004 and December 31, 2008. Baseline data were collected at the most recent diagnosis of tuberculosis and mortality was assessed between March and May of 2014. We calculated standardized mortality ratios (SMR) and case-fatality rates for all participants and for subgroups. Univariate and multivariate Cox regression models were used to quantify associations between co-morbidities and mortality from all causes and from tuberculosis.

**Findings:**

We registered 4569 subjects in the cohort. Overall, the cohort had an SMR for deaths from all causes of 5.2 (95% confidence interval, CI: 4.8–5.6). Males had a higher SMR than females (6.1 versus 3.0). After adjustment for age and sex, hazard ratios (HR) for deaths from all causes were significantly greater in previously treated people (HR: 1.26; 95% CI: 1.08–1.49) and sputum smear-test positive people (HR: 1.55; 95% CI: 1.35–1.78). The risk of death from tuberculosis was also significantly greater for previously treated people (HR: 1.88; 95% CI: 1.24–2.86) and smear positive people (HR: 3.16; 95% CI: 2.06–4.87).

**Conclusion:**

People with tuberculosis in Shanghai have an increased risk of mortality. Earlier diagnosis and more vigilant follow-up may help to reduce mortality in this group.

## Résumé

**Objectif:**

Déterminer la surmortalité dans une cohorte de personnes atteintes de tuberculose à Shanghai.

**Méthodes:**

Les participants étaient des habitants de 4 districts (sur 19) de Shanghai, enregistrés dans une des quatre cliniques de la tuberculose entre le 1er janvier 2004 et le 31 décembre 2008. Des données de référence ont été recueillies lors du diagnostic le plus récent de tuberculose et la mortalité a été évaluée entre mars et mai 2014. Nous avons calculé les rapports standardisés de mortalité (RSM) et les taux de létalité pour tous les participants ainsi que pour des sous-groupes. Des modèles de régression univariée et multivariée de Cox ont été utilisés pour quantifier les associations entre les comorbidités et la mortalité, toutes causes confondues et due à la tuberculose.

**Résultats:**

Nous avons enregistré 4569 sujets dans la cohorte. Globalement, la cohorte avait un RSM pour les décès toutes causes confondues de 5,2 (intervalle de confiance (IC) de 95%: 4,8-5,6). Les hommes affichaient un RSM plus élevé que les femmes (6,1 contre 3,0). Après ajustement en fonction de l'âge et du sexe, les ratios de risque (RR) de décès toutes causes confondues étaient nettement plus élevés chez les personnes ayant précédemment reçu un traitement (RR: 1,26; IC 95%: 1,08–1,49) et les personnes à frottis d'expectoration positif (RR: 1,55; IC 95%: 1,35-1,78). Le risque de décès dû à la tuberculose était également beaucoup plus important pour les personnes ayant précédemment reçu un traitement (RR: 1,88; IC 95%: 1,24-2,86) et les personnes à frottis positif (RR: 3,16; IC 95%: 2,06-4,87).

**Conclusion:**

Les personnes atteintes de tuberculose à Shanghai présentent un risque accru de mortalité. Un diagnostic plus précoce et un suivi plus attentif pourraient permettre de réduire la mortalité dans ce groupe.

## Resumen

**Objetivo:**

Determinar el exceso de mortalidad en una cohorte de personas con tuberculosis en Shanghái.****

**Métodos:**

Los participantes eran residentes locales de 4 (de 19) distritos de Shanghái, registrados en uno de los cuatro consultorios antituberculosos entre el 1 de enero de 2004 y el 31 de diciembre de 2008. Se recogieron datos de referencia de los diagnósticos de tuberculosis más recientes y se evaluó la mortalidad entre marzo y mayo de 2014. Se calcularon las tasas de mortalidad estandarizadas (SMR) y la tasa de letalidad de todos los participantes y por subgrupos. Se utilizaron modelos de regresión de Cox univariables y multivariables para cuantificar las asociaciones entre la comorbilidad y la mortalidad de todas las causas de tuberculosis.

**Resultados:**

Se registraron 4.569 sujetos en la cohorte. En general, la cohorte tenía una SMR para muertes de todas las causas de 5,2 (intervalo de confianza del 95%, IC: 4,8–5,6). Los hombres tenían una SMR mayor que las mujeres (6,1 frente a 3,0). Tras ajustarlos de acuerdo a edad y sexo, los cocientes de riesgo (HR) para las muertes de todas las causas eran significantemente mayores en personas tratadas con anterioridad (HR: 1,26 (IC del 95%: 1,08–1,49) y personas con pruebas de esputo positivas (HR: 1,55 (IC del 95%: 1,35–1,78). El riesgo de muerte por tuberculosis también era significativamente mayor en personas tratadas con anterioridad (HR: 1,88 (IC del 95%: 1,24-2,86) y personas con pruebas de esputo positivas (HR: 3,16 (IC del 95%: 2,06–4,87).

**Conclusión:**

Las personas con tuberculosis en Shanghái tienen un mayor riesgo de mortalidad. Unos diagnósticos más tempranos y un seguimiento más atento podrían ayudar a reducir la mortalidad de este grupo.

## ملخص

**الغرض:**

تحديد الزيادة في حالات الوفاة في مجموعة من المصابين بالسل في شانغهاي.

**الطريقة:**

كان المشاركون من السكان المحليين في 4 أحياء (من بين 19 حيًا) في شنغهاي، وقد تم تسجيلهم في واحدة من أربع عيادات لعلاج السل في الفترة بين 1 يناير/كانون الثاني 2004 و31 ديسمبر/كانون الأول 2008. وقد تم جمع البيانات الأساسية على مستوى أحدث الحالات التي تم تشخيصها كحالات الإصابة بالسل، كما تم تقدير الوفيات في الفترة بين شهري مارس/آذار ومايو/أيار 2014. وحسبنا نسب الوفيات القياسية (SMR) ومعدلات الوفيات بين الحالات المصابة بالنظر إلى جميع المشاركين والمجموعات الفرعية. ****وتم استخدام نماذج انحدار كوكس ذات المتغير الأحادي والمتعددة المتغيرات لقياس مدى الارتباط بين الإصابة بأكثر من مرض في نفس الوقت وحالات الوفاة الناتجة عن جميع الأسباب والناتجة عن مرض السل.

**النتائج:**

سجلنا 4569 من الحالات محل الدراسة في المجموعة المشار إليها. ****وبوجه عام، بلغت نسبة الوفيات القياسية الناتجة عن جميع الأسباب في هذه المجموعة 5.2 (بنسبة أرجحية مقدارها ‏‏95%: 4.8–5.6). وارتفعت نسبة الوفيات القياسية بين الرجال مقارنةً بالنساء (6.1 مقابل 3.0). ****وبعد تعديل عامل العمر والجنس، كانت نسب خطورة (HR) التعرض للوفاة الناتجة عن جميع الأسباب أكثر ارتفاعًا بشكل ملحوظ لدى الأشخاص الذين سبقت معالجتهم (نسبة خطورة: 1.26؛ بنسبة أرجحية مقدارها ‏95%: 1.08– 1.49) ولدى الأشخاص الذين كانت نتائج اختبار مسحة البلغم الخاصة بهم إيجابية (HR: 1.55؛ بنسبة أرجحية مقدارها ‏95%: 1.35–1.78). وكان خطر الوفاة الناتجة عن السل أكثر ارتفاعًا أيضًا بشكل ملحوظ لدى الأشخاص الذين سبقت معالجتهم (نسبة خطورة: 1.88؛ بنسبة أرجحية مقدارها 95‏%: 1.24– 2.86) ولدى الأشخاص الذين كانت نتائج المسحة الخاصة بهم إيجابية (نسبة خطورة: 3.16؛ بنسبة أرجحية مقدارها ‏95%: 2.06–4.87) **. **

**الاستنتاج:**

يعاني المصابون بالسل في شانغهاي من زيادة خطر التعرض للوفاة. وقد يساعد التشخيص المبكر للمرض والمزيد من المتابعة الطبية الدقيقة في تقليل معدل الوفيات في هذه المجموعة.

## 摘要

**目的:**

旨在确定上海地区肺结核病患者的超额死亡率。

**方法:**

参与者是曾于2004 年 1 月 1 日至 2008 年 12 月 31 日在四家肺结核病诊所其中一家登记过的、上海市 4 个区（共 19 个区）的当地居民。我们收集了最近的肺结核病诊断的基线数据，并对 2014 年 3 月至 5 月的死亡率进行了评估。我们计算出所有参与者和子组的标准化死亡率和病死率，并采用单变量和多变量 Cox 回归模型量化各种原因与肺结核导致的共病症与死亡率之间的关联。

**结果:**

我们登记了病患中 4569 名受试者。总体而言，各种原因导致的死亡患者的标准化死亡率为 5.2 （95% 置信区间：4.8–5.6）。男性的标准化死亡率高于女性 (6.1 比 3.0)。调整年龄和性别后，以前接受过治疗的患者（风险比：1.26，95% 置信区间：1.08-1.49）和痰涂片试验呈阳性的患者（风险比：1.55，95% 置信区间：1.35-1.78）中各种原因导致的死亡风险比明显更高。以前接受过治疗的患者（风险比率：1.88，95% 置信区间：1.24-2.86）和痰涂片试验呈阳性的患者（风险比率：3.16，95% 置信区间：2.06-4.87）中肺结核导致的死亡风险也明显更高。

**结论:**

上海地区肺结核病患者的死亡率风险增加。及早的诊断和更加警觉的随访诊查可能有助于降低肺结核患者的死亡率。

## Резюме

**Цель:**

Определение повышенной смертности в когорте больных туберкулезом, проживающих в г. Шанхай.

**Методы:**

В исследовании приняли участие местные жители 4 районов Шанхая (из 19), зарегистрированные в одной из четырех туберкулезных клиник в период с 1 января 2004 года по 31 декабря 2008 года. Сбор исходных данных осуществлялся при последнем диагностировании туберкулеза, а оценка смертности проводилась в период с марта по май 2014 года. Были рассчитаны стандартизированные показатели смертности (SMR) и показатели летальности для всех участников и подгрупп. Для количественного определения корреляции между сопутствующими заболеваниями и смертностью по всем причинам и от туберкулеза были использованы одновариантная и многовариантная модели регрессии Кокса.

**Результаты:**

В когорте было зарегистрировано 4569 субъектов. В целом по когорте SMR для смерти от всех случаев составил 5,2 (95%-й доверительный интервал, ДИ: 4,8–5,6). Для мужчин SMR был выше, чем для женщин (6,1 против 3,0). С учетом поправки на возраст и пол относительный риск (ОР) для смерти по всем причинам был значительно выше для лиц, ранее получавших лечение (ОР: 1,26; 95%-й ДИ: 1,08–1,49), и для лиц с положительным результатом микроскопии мазка мокроты (ОР: 1,55; 95%-й ДИ: 1,35–1,78). Риск смерти от туберкулеза был также значительно выше для лиц, ранее получавших лечение (ОР: 1,88; 95%-й ДИ: 1,24–2,86), и для лиц с положительным результатом микроскопии мазка (ОР: 3,16; 95%-й ДИ: 2,06–4,87).

**Вывод:**

Для лиц с туберкулезом, проживающих в Шанхае, существует повышенный риск смертности. Ранняя диагностика и более строгий последующий контроль могут способствовать снижению смертности в данной группе лиц.

## Introduction

China has approximately 1 million new cases of tuberculosis per year,^1^^–^^3^ resulting in a substantial burden of premature mortality.^2^ Several factors are known to increase the risk of tuberculosis-associated mortality, including drug resistance, disease severity, irregular or incomplete treatment, human immunodeficiency virus (HIV) infection, smoking and alcoholism.^4^ Multidrug-resistant (MDR) tuberculosis poses a major threat to tuberculosis control. A national survey done in 2008 found that 5.7% of people newly diagnosed with tuberculosis, and 25.6% of those who had previously been treated, had MDR-tuberculosis.^5^ In Shanghai, China, people being treated for tuberculosis had a case-fatality rate (CFR) of 5.5% in 2008,^6^ and in 2010 another national survey reported a CFR of 5.1%.^7^ The purpose of the present study was to determine the mortality rate and excess mortality in a cohort of people with tuberculosis who were registered in four districts of Shanghai from 2004 to 2008 and to identify groups in this cohort at high risk of death.

## Methods

The study sample consisted of local residents from four districts in Shanghai City: Yangpu, Pudong, Putuo and Songjiang, with a total population of 9.23 million in 2014. The districts were chosen based on geographic location and tuberculosis notifications. Our study population consisted of 5001 local participants who were registered in tuberculosis clinics under the national tuberculosis programme between January 1, 2004 and December 31, 2008. We included both newly diagnosed and previously treated participants.

Beginning in the 1990s, the national tuberculosis programme implemented a mandatory reporting system for people with tuberculosis in Shanghai. Each person with suspected tuberculosis who seeks health care in facilities in Shanghai is referred to a specialized tuberculosis hospital or clinic where chest X-rays, sputum smears and cultures are done to confirm the diagnosis. Three sputum specimens are routinely collected from each person. People with bacteriological confirmation or abnormal chest X-ray results are routinely treated at a tuberculosis reference hospital or clinic. All *M. tuberculosis* isolates were sent to the tuberculosis reference laboratory at the Shanghai Center for Disease Control and Prevention (CDC) or to the Shanghai Pulmonary Hospital for drug susceptibility testing.

The ethics committee of the School of Public Health of Fudan University approved the study. All participants provided written informed consent to allow their information to be stored and used for research. The study was a sub-study conducted within a larger underlying study.

### Definitions

An isolate was considered as MDR-tuberculosis if it was resistant to both isoniazid and rifampin. Cause of death was based on information in the death certificate and classified according to the *International Statistical Classification of Diseases and Related Health Problems, 10th Revision* (ICD-10).^8^ Deaths among people with both HIV and tuberculosis are classified as deaths from HIV in ICD-10. Due to a low percentage of HIV-positive people in the study population (0.28–3.30%),^9^^,^^10^ all participants were assumed to be HIV-negative. The case-fatality rate is defined as the risk of death from tuberculosis among people diagnosed with active tuberculosis.^11^

Mortality was measured as: (i) the standardized mortality ratio (SMR; see Box 1); and (ii) the case-fatality rate at 1, 5 and 10 years from the start of treatment. The case-fatality rate was estimated as the number of deaths divided by the total number of people with active tuberculosis.

Box 1 **Estimating tuberculosis mortality**

In 2002, the World Health Organization (WHO) defined any death of a tuberculosis patient during treatment as attributable to tuberculosis, irrespective of the final cause of death.^12^ As a result, several recent studies used all-cause mortality as a surrogate marker of mortality attributable to tuberculosis.^13^

Since 2013, WHO Global Tuberculosis Reports have defined mortality from tuberculosis as any death caused by tuberculosis in HIV-negative individuals.^2^ When reported as a rate, tuberculosis mortality has typically been expressed as a person-time mortality rate or more commonly as a case-fatality rate (the risk of death from tuberculosis among individuals with active tuberculosis) within a specific time period. However, the case-fatality rates reported for tuberculosis, which range from 12% to 44%, cannot be compared among studies because they were determined as cumulative mortality using different follow-up durations.^4^^,^^14^ In addition, the tuberculosis mortality rate is affected by the baseline mortality rate of the study population.^13^^,^^15^

A better, though indirect, measure of tuberculosis mortality is the standardized mortality ratio (SMR). The SMR is defined as the observed mortality in people with tuberculosis relative to the expected mortality based on the age-specific mortality rates in a standard population. We used the national population of China in 2013 as our standard population.^16^

### Baseline survey

Inclusion criteria were as follows: registered in a tuberculosis clinic under the national tuberculosis programme between January 1, 2004 and December 31, 2008; having household registration or continuous residence at the study site for at least 6 months in the previous year; and provision of written informed consent by the participants or their relatives. Baseline data were collected at the most recent diagnosis of tuberculosis (between 2002 and 2008) and included name, age, sex, residential address, category of treatment and date of registration from the national tuberculosis programme register. Comorbidities, behavioural risk factors (such as smoking) and other data were extracted from paper copies of medical records.

### Follow-up

From March to May 2014, health workers visited the households of all participants at least once and interviewed participants or their close relatives who lived in the same household. Deaths were reported by household members and mortality data, including date of death, were collected from death certificates. For quality control, 10% of subjects were re-interviewed; trained public health workers checked data by telephone or in direct visits. Participants were followed up for a range of 1886 to 5205 days (5.17 to 10.67 years), starting from registration to the last follow-up in 2014 or until the date of death.

### Regression analysis

Univariate and multivariate Cox regression models were used to identify significant co-morbidities during treatment for tuberculosis that were associated with all-cause mortality in SPSS statistical software version 16.0 (SPSS Inc., Chicago, United States of America). Hazard ratios (HR), 95% confidence intervals (CI), and *P*-values were calculated to assess the significance of associations.

## Results

A total of 5001 participants met our inclusion criteria (Table 1). Of these, 432 were excluded from analysis because they were misdiagnosed, declined to participate in the follow-up, relocated to a district without a tuberculosis register or failed to provide a new address. Of 4569 participants with successful follow-up (91.4%), 3396 were men (74.3%) and 1173 were women (25.7%; Table 1). Among the participants, 3601 (78.8%) survived the entire follow-up period from the start of treatment and 968 (21.2%) died. Men were more likely than women to have lung cavitation (29.4% versus 20.3%; *P* < 0.001), two or more comorbidities (3.0% versus 1.5%, *P* = 0.008) and be smear-positive (48.0% versus 39.1%, *P* < 0.001), but were less likely to have received previous treatment (10.9% versus 35.5%, *P* < 0.001).

**Table 1:** Mortality in tuberculosis patients, Shanghai, China, 2004–2014

Group	No.	Person-years follow-up	Observed deaths	Mortality rate per 1 000 person-years	Expected deaths^a^	SMR^b^ (95% CI)^c^
**All**	4569	29 744	968	32.5	282.7	5.2 (4.8–5.6)
**Age group, years**						
0–19	46	301	0	0.0	0.6	0.0 (0.0–2.0)
20–39	1007	7471	15	2.0	5.0	0.3 (0.2–0.5)
40–59	1771	12 500	175	14.0	58.6	2.3 (1.9–2.65)
60–79	1212	7280	411	56.5	285.8	9.1 (8.2–10.0)
≥ 80	525	2101	363	172.8	401.4	27.9 (25.1–30.9)
Unknown	8	46	4	87.9	0.5	14.2 (3.9–36.3)
**Sex**						
Male	3396	21 628	819	37.9	236.9	6.1 (5.7–6.5)
Female	1173	8116	149	18.4	62.9	3.0 (2.5–3.5)
**Diagnosis**						
Secondary pulmonary	4108	26 698	882	33.0	254.7	5.3 (5.0–5.7)
Primary pulmonary	50	332	3	9.0	3.1	1.5 (0.3–4.3)
Disseminated pulmonary	15	70	8	114.8	0.9	18.5 (8.0–36.5)
Extra-pulmonary	367	2475	71	28.7	22.8	4.6 (3.6–5.8)
Other	29	169	4	23.6	1.8	3.8 (1.0–9.8)
**Treatment management**						
Directly-observed	1152	7702	227	29.5	71.4	4.8 (4.2–5.4)
Self-administered	30	261	4	15.3	1.9	2.5 (0.7–6.3)
Hospitalized	1907	11 922	504	42.3	118.2	6.8 (6.2–7.4)
Family-observed	1250	8491	197	23.2	77.5	3.7 (3.2–4.3)
Unknown	230	1368	36	26.3	14.3	4.2 (3.0–5.9)
**Previously treated**						
Yes	785	4953	202	40.8	48.7	6.6 (5.7–7.6)
No	3784	24 791	706	28.5	234.6	4.6 (4.3–4.9)
**MDR-tuberculosis**						
Yes	41	199	17	85.5	2.5	13.8 (8.0–22.1)
No	1449	9671	354	36.6	89.8	5.9 (5.3–6.6)
Unknown	3079	19 874	597	30.0	190.9	4.8 (4.5–5.2)
**Sputum smear test**						
Yes	2341	12 602	334	26.5	145.1	4.3 (3.8–4.8)
No	2089	16 189	616	38.0	129.5	6.1 (5.7–6.6)
Unknown	139	952	18	18.9	8.6	3.0 (1.8–4.8)
**Cavitation**						
Yes	1235	7892	257	32.6	203.2	5.3 (4.6–5.9)
No	3277	21 526	691	32.1	76.6	5.2 (4.8–5.6)
Unknown	57	326	20	61.4	3.5	9.9 (6.0–15.3)
**Comorbidity**						
Diabetes	473	2864	129	45.0	29.3	7.3 (6.1–8.6)
COPD	22	100	14	140.0	1.4	22.6 (12.3–37.9)
Hypertension	67	385	28	72.8	4.2	11.7 (7.8–17.0)
Chronic bronchitis	87	418	46	110.0	5.4	17.7 (13.0–23.7)
Cancer	36	134	25	186.8	2.2	30.1 (19.5–44.5)

CI: confidence interval; COPD: chronic-obstructive pulmonary disease; MDR: multidrug-resistant; SMR: standardized mortality ratio.

^a^ Calculated by multiplying the number of people with tuberculosis and age specific mortality rates of general population.^16^

^b^ Observed divided by expected mortality.

^c^ Calculated assuming a Poisson distribution.

The overall SMR was 5.2 (95% CI: 4.8–5.6); the SMR increased with age in our cohort. Among participants who were 20–39 years-old, the SMR was 0.3 (i.e. lower than the general population), but the SMR increased to 27.9 for participants older than 79 years. Men had a higher SMR than women (6.1 versus 3.0). Participants with disseminated pulmonary disease had a higher SMR (18.5) than those with primary pulmonary disease (1.5) and secondary pulmonary disease (5.3). Previously treated participants had a higher SMR than participants who were undergoing their first treatment for tuberculosis (6.6 versus 4.6). Participants with comorbidities also had high SMRs, especially those with chronic obstructive pulmonary disease (COPD) (22.6) and cancer (30.1).

The 1, 5 and 10 year case-fatality rates were 7.48%, 17.20%, and 21.23%, respectively (Table 2). Participants with disseminated pulmonary disease had the highest 1 year case-fatality rate (40.00%), whereas those with cancer had the highest 5 year case-fatality rate (61.11%). The 5 year case-fatality rate was also high for participants older than 79 years (58.89%), with COPD (45.45%), chronic bronchitis (44.83%) or MDR-tuberculosis (39.02%). At the 10 year follow up, the case-fatality rate was highest for participants with cancer (69.44%) or COPD (63.64%) and was also high for those aged 80 years and older (69.02%).

**Table 2:** Cumulative case-fatality rates in tuberculosis patients, Shanghai, China, 2004–2014

Subgroup	No.	Cumulative case-fatality rate, (%)		
		1 year	5 years	10 years
**All**	4569	7.48	17.20	21.23
**Age group, years**				
0–19	46	0.00	0.00	0.00
20–39	1007	0.40	1.09	1.39
40–59	1771	3.12	8.56	9.59
60–79	1212	10.96	25.75	33.89
≥ 80	525	28.49	58.89	69.02
Unknown	8	12.50	50.00	50.00
**Sex**				
Male	3396	8.73	19.73	23.96
Female	1173	3.93	10.00	12.48
**Diagnosis**				
Secondary pulmonary	4108	7.45	17.60	21.28
Primary pulmonary	50	4.00	6.00	6.00
Disseminated pulmonary	15	40.00	46.67	53.33
Extra-pulmonary	367	6.81	12.81	19.35
Other	29	6.90	13.79	13.79
**Clinical management**				
Directly-observed	1152	5.47	15.36	19.44
Self-administered	30	0.00	6.67	13.33
Hospitalized	1907	11.12	21.97	26.17
Family-observed	1250	4.32	12.24	15.44
Unknown	230	5.22	14.35	15.65
**Previously treated**				
Yes	785	8.54	20.25	25.48
No	3784	7.24	16.52	19.98
**MDR-tuberculosis**				
Yes	41	12.20	39.02	41.46
No	1449	6.69	18.91	24.15
Unknown	3079	7.76	16.04	19.13
**Sputum smear test**				
Yes	2341	9.95	21.87	26.01
No	2089	4.93	12.40	15.75
Unknown	139	3.60	9.35	12.95
**Cavitation**				
Yes	1235	20.16	44.78	55.47
No	3277	2.59	6.53	7.66
Unknown	57	12.28	29.82	35.09
**Comorbidity**				
Diabetes	473	9.51	21.99	26.85
COPD	22	18.18	45.45	63.64
Hypertension	67	8.96	29.85	41.79
Chronic bronchitis	87	25.29	44.83	52.87
Cancer	36	36.11	61.11	69.44

COPD: chronic-obstructive pulmonary disease; MDR: multidrug-resistant.

Table 3 shows the hazard ratios (HR) for deaths from all causes and from tuberculosis, with and without adjustment for age and sex. The adjusted HRs for deaths from all causes were 1.50 (95% CI: 0.91–2.48) for participants with MDR-tuberculosis, 1.55 (95% CI: 1.35–1.78) for smear-positive participants, and 1.26 (95% CI: 1.08–1.49) for previously treated participants. The adjusted HRs for deaths from tuberculosis were 1.77 (95% CI: 0.62–5.06) in participants with MDR-tuberculosis, 3.16 (95% CI: 2.06–4.87) for smear-positive participants and 1.88 (95% CI: 1.24–2.86) for previously treated participants. We also calculated the adjusted HRs for participants with diabetes (0.97; 95% CI: 0.80–1.17), COPD (1.47; 95% CI: 0.86–2.50), hypertension (1.43; 95% CI: 0.84–2.46), chronic bronchitis (1.42; 95% CI: 1.05–1.94) and cancer (1.93; 95% CI: 1.29–2.90).

**Table 3:** Crude and adjusted hazard ratios for mortality in tuberculosis patients, Shanghai, China, 2004–2014

Subgroup	HR (mortality from tuberculosis)			HR (mortality from all causes)	
	Crude	Adjusted (95% CI)^a^		Crude	Adjusted (95% CI)^a^
**Diagnosis**					
Secondary pulmonary	1.00	1.00		1.00	1.00
Primary pulmonary	0.66	1.42 (0.19–10.39)		0.28	0.68 (0.22–2.13)
Disseminated pulmonary	2.31	1.86 (0.26–13.51)		3.38	2.10 (1.04–4.24)
Extra-pulmonary	0.72	0.68 (0.33–1.40)		0.84	0.81 (0.63–1.05)
Other	1.58	1.46 (0.20–10.65)		0.69	0.85 (0.32–2.29)
**Clinical management**					
Directly-observed	1.00	1.00		1.00	1.00
Self-administered	–	–		0.57	1.22 (0.45–3.34)
Hospital	1.80	2.09 (1.18–3.73)		1.43	1.39 (1.14–1.69)
Family-observed	0.64	0.97 (0.47–1.98)		0.79	0.99 (0.79–1.25)
Unknown	1.70	1.76 (0.64–4.87)		0.87	0.88 (0.57–1.35)
**Previously treated**					
Yes	1.67	1.88 (1.24–2.86)		1.31	1.26 (1.08–1.49)
No	1.00	1.00		1.00	1.00
**MDR-tuberculosis**					
Yes	2.79	1.77 (0.62–5.06)		2.12	1.50 (0.91–2.48)
No	1.00	1.00		1.00	1.00
Unknown	0.65	0.72 (0.48–1.08)		0.81	0.95 (0.82–1.11)
**Sputum smear test**					
Yes	4.33	3.16 (2.06–4.87)		2.31	1.55 (1.35–1.78)
No	1.00	1.00		1.00	1.00
Unknown	1.21	1.34 (0.32–5.67)		0.93	0.97 (0.60–1.56)
**Cavitation**					
Yes	1.28	1.32 (0.91–1.93)		0.99	1.04 (0.90–1.20)
No	1.00	1.00		1.00	1.00
Unknown	2.74	2.69 (0.97–7.45)		1.84	1.59 (1.02–2.49)
**Comorbidity**					
Diabetes	1.09	0.85 (0.49–1.46)		1.40	0.97 (0.80–1.17)
COPD	1.58	0.88 (0.12–6.37)		4.05	1.47 (0.86–2.50)
Hypertension	0.50	0.28 (0.04–2.03)		5.29	1.43 (0.84–2.46)
Chronic bronchitis	3.00	2.03 (0.91–4.54)		3.33	1.42 (1.05–1.94)
Cancer	1.96	1.26 (0.31–5.16)		5.10	1.93 (1.29–2.90)

CI: confidence interval; COPD: chronic obstructive pulmonary disease; HR: hazard ratios; MDR: multidrug resistant.

^a^ Based on Cox regression models, adjusted for age and sex.

**Fig. 1:**
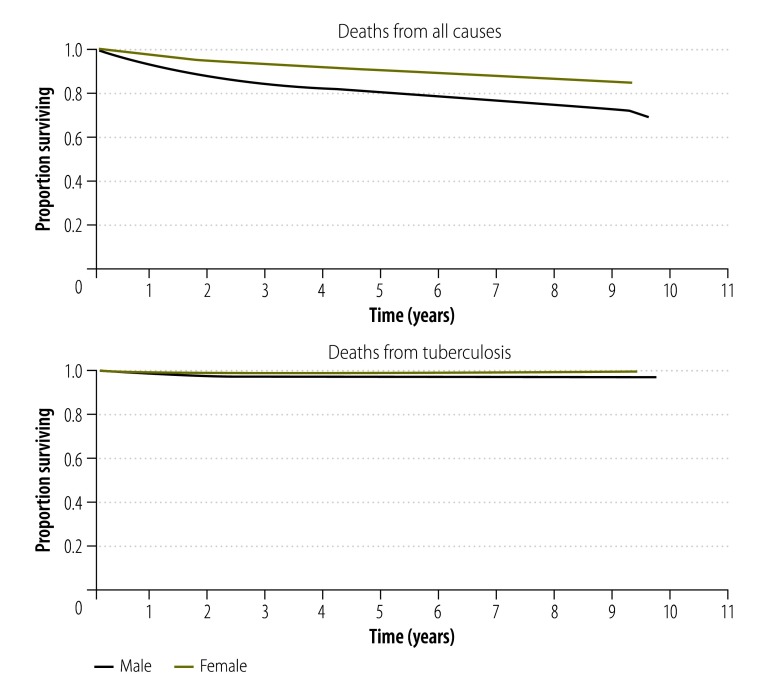
Survival curves for study participants, Shanghai, China, 2004–2014

The cumulative survival curves (Fig. 1) show that the highest risk of death was in the first year, especially during the 2-month intensive treatment phase. After the treatment period, survival improved, especially when considering deaths from tuberculosis only.

## Discussion

The risk of death in the study population was five times that in the general population of China (SMR 5.2). This is lower than previously reported for people with tuberculosis in India (6.1),^17^ the Netherlands (8.3),^18^ and Ethiopia (10.0),^19^ at least in part because there is less co-infection with HIV in China.^20^ Men had higher mortality rates than women, which may be because of sex differences in clinical characteristics (smear results, cavitation, prevalence of comorbidities and MDR-tuberculosis),^21^ a higher likelihood of noncompliance with treatment, or the presence of additional risk factors such as smoking.^6^^,^^17^^,^^19^^,^^22^ Participants with disseminated pulmonary disease, smear-positive disease or MDR-tuberculosis had higher SMRs than those without these characteristics. This is as expected, given that all of these characteristics are related to disease severity.

Among our participants, 25% were undergoing directly observed treatment, short-course (DOTS) facilitated by health-care workers. Contrary to the general expectation that DOTS will substantially increase the effectiveness of treatment for tuberculosis,^23^^,^^24^ we found that participants receiving DOTS facilitated by health-care workers had a higher cumulative case-fatality rate than those under self-administered and family-observed management. This may be because people who were willing to remain under DOTS were in poorer health than those under self-administered and family-observed management. Consistent with our findings for Shanghai, studies in Ethiopia,^25^ India^26^ and the United Republic of Tanzania^27^ also reported good treatment outcomes following self-administered tuberculosis treatment. However, contrary results have been obtained in other settings. For example, a study of health-community workers found that DOTS was more beneficial than family-observed management,^28^ and a randomized trial in Nepal indicated that family-observed management produced similar outcomes as health-community worker facilitated DOTS.^29^ Hospitalized people with tuberculosis in China typically have severe disease, which is consistent with this group having the highest cumulative case-fatality rate in our cohort.^30^

Mortality was highest in the first year, and then declined substantially with time in almost all of the analysed subgroups. During the 10 year follow-up, about one third of the deaths occurred in the first year, and 80% of the deaths occurred within five years of diagnosis. Tuberculosis-related mortality continued to occur even after the completion of treatment. This emphasizes that the definition of tuberculosis mortality should not be restricted to the treatment period alone, because this may lead to an underestimation of tuberculosis-related mortality.

Previous studies have identified several co-morbidities that are risk factors for all-cause mortality during tuberculosis treatment, including renal failure, respiratory disease, cardiovascular disease, cancer, COPD and diabetes.^31^^–^^33^ Our analysis identified several such comorbidities. In univariate analyses, people with tuberculosis with diabetes, COPD, chronic bronchitis, hypertension and cancer also had increased mortality. However, after adjusting for age and sex, only chronic bronchitis and cancer were significantly associated with death from all causes. The impact of chronic bronchitis on all-cause mortality is consistent with our previous report.^15^ It is possible that damage from chronic bronchitis may exacerbate some of the symptoms of tuberculosis, as both conditions are associated with chronic airflow obstruction and other respiratory symptoms.^34^

Our study has several limitations. First, we did not ascertain the HIV status of participants because HIV testing is not compulsory for people with tuberculosis in China. However, previous screening studies reported that the HIV prevalence among people with tuberculosis in China was low.^9^^,^^10^ Second, we determined the cause of death using the death certificate database. In Shanghai, a death registration system was established based on ICD-10 for defining the causes of death. Thus, there may have been misclassification of tuberculosis deaths, although misclassification bias would have been reduced by our policy of confirming cause of death during follow-up. Participants who self-administered treatment were possibly marked as being under DOTS in the tuberculosis management system,^35^ which may have introduced a bias. Loss to follow-up during or after treatment may have led to an overestimation of mortality rates, since recording of deaths from the death registration database is nearly complete. Finally, although geographic characteristics and tuberculosis prevalence were considered in selecting the study districts, the selected districts may not have provided a representative sample of the population of Shanghai.

## Conclusion

In this cohort of people with tuberculosis in Shanghai, mortality was higher during treatment, suggesting the importance of improving clinical management and treatment for tuberculosis. Interventions during treatment (i.e. monitoring and managing the side-effects of anti-tuberculosis medication) may reduce the rate of tuberculosis deaths while follow-up can lead to reduced deaths from other causes. Timely detection and management of comorbidities among people with tuberculosis is necessary to prevent deaths during treatment for tuberculosis, as reported by other studies.^36^ People with tuberculosis and major comorbidities such as chronic bronchitis and lung cancer need careful management. When appropriate, follow-up and assessment coordinated by tuberculosis departments may improve the management of these conditions. Post-treatment mortality could be used as additional evidence of case fatality (obtained through routine reports) to better characterize overall mortality in people with tuberculosis.
